# Chatbots and beyond: transforming cannabis use interventions with innovative AI approaches

**DOI:** 10.3389/ijph.2026.1609866

**Published:** 2026-10-26

**Authors:** Yuqi Hu

**Affiliations:** Herbert Wertheim School of Public Health and Human Longevity Science, University of California San Diego, La Jolla, CA, United States

**Keywords:** artificial intelligence, cannabis use, chatbots, just-in-time adaptive interventions, psychosocial intervention

**The IJPH series “Young Researcher Editorial” is a training project of the Swiss School of Public Health**

Cannabis use and dependence is a rising public health challenge worldwide. Globally, the number of people with cannabis use disorder and its associated burden of diseases have notably increased in the past decades, particularly affecting young people [1, 2]. Despite this rising burden, public health responses remain limited.

While there are no established pharmacological treatments for cannabis use and dependence, psychosocial approaches, including motivational and cognitive-behavioral interventions, may improve cannabis use outcomes [3]. However, access to these treatments remains limited by workforce, time and transportation barriers. Treatment-related stigma, shaped by histories of drug control and enforcement that unevenly affect underserved populations, also discourages formal help-seeking [4]. Moreover, existing interventions are rarely delivered when people need support most and are often minimally responsive to changing risk states and individual needs. In this context, recent advances in artificial intelligence (AI) create an opportunity to address these gaps by making cannabis interventions more accessible, scalable, timely, and personalized.

AI conversational chatbots are one promising approach. In practice, chatbots could be engineered to deliver core elements of psychosocial interventions for cannabis use by helping users set goals, identify triggers, strengthen motivation, and practice coping and relapse-prevention skills. A recent systematic review of chatbot-assisted substance use interventions, mainly for tobacco and alcohol use, found that experimental studies reported meaningful effects on substance use outcomes and users generally found the interventions useful and acceptable [5]. Importantly, chatbots could address existing access gaps by providing private, on-demand support outside conventional clinic settings and extending services to people facing geographic, transportation, scheduling, or stigma-related barriers. They could scale up at exceptionally low marginal costs, reducing reliance on scarce trained professionals.

AI could also improve the timeliness of cannabis use interventions through just-in-time adaptive interventions (JITAIs). JITAIs can use machine learning methods to identify moments of heightened vulnerability and trigger support in a timely manner, although evidence of their effectiveness for substance use remains limited [6]. For cannabis use, this could involve drawing on self-reported data, prior engagement patterns, time, location, or other digital signals to identify vulnerable moments, such as craving after work, social exposure to use, boredom at night, or stress after conflict. The intervention could then provide a real-time response, such as a motivational message, coping prompt, or brief cognitive-behavioral exercise. Because cannabis use risk is often episodic and context-dependent, JITAIs could complement psychosocial approaches by delivering their content at high-risk moments rather than only in scheduled or user-initiated sessions.

Conversational chatbots and JITAIs could be combined in intervention design. In such a model, the JITAI component identifies moments of heightened risk using self-reported, behavioral, or contextual data, while the chatbot delivers support in a conversational format, which could be personalized to adapt to the user’s current state. For example, a recent smoking-cessation intervention integrated JITAI with a generative chatbot and was evaluated favorably by a domain expert [7].

While direct evidence remains limited, certain elements of psychosocial interventions for tobacco use may be adaptable to cannabis interventions, as these substances overlap in psychosocial processes such as motivation, cravings, triggers, coping, and relapse. This possibility is supported by evidence that cannabis co-users who reduced cigarette use in a smoking cessation intervention may concurrently reduce their cannabis use, even though cannabis use was not explicitly addressed [8]. Therefore, existing evidence on tobacco interventions [5, 7] could support the feasibility of AI-enabled cannabis use interventions. Nevertheless, adaptation would be required to account for the unique characteristics of cannabis use, including diverse products and routes of administration, self-medication, associations with social identity, and legal status.

Above all, innovative AI interventions for cannabis use must be designed with safety and privacy as core requirements. A review found over half of health-related chatbots failed to respond adequately to high-risk messages such as suicidal thoughts [9]. Such limitations may become particularly consequential in cannabis interventions, where users may experience psychotic symptoms or other crises. AI systems also raise privacy concerns because they may collect sensitive behavioral data, infer vulnerability, and transmit information across systems that users do not fully understand [10]. Depending on the jurisdiction, intervention data may be subject to compelled disclosure in legal or administrative proceedings. Systems should therefore have a clearly limited clinical scope, minimize data collection and retention, clearly explain any limits of confidentiality, and provide reliable escalation pathways to human support.

In summary, AI approaches could substantially extend the reach of psychosocial support for cannabis use and warrant more sustained research. Design, development and evaluation of innovative AI interventions should extend beyond a focus on efficacy to considerations of whether these tools improve engagement and reduce access and stigma-related barriers without safety or privacy concerns. Future implementation research should identify which populations benefit or remain underserved, establish reliable pathways to human care, and examine how effective innovations can be integrated into routine care and public health infrastructure.

## Data Availability

The original contributions presented in the study are included in the article/supplementary material, further inquiries can be directed to the corresponding author.
